# Tailoring treatment for elderly bladder cancer: a case report of personalized management of high-grade urothelial carcinoma with papillary features

**DOI:** 10.3389/fonc.2024.1434795

**Published:** 2024-08-06

**Authors:** Christos G. Nikolaidis, Despoina Gyriki, Charalambos Anitsakis, Elisavet Stavropoulou

**Affiliations:** ^1^ Internal Medicine Department, Vostaneio-General Hospital of Mytilene, Mytilene, Greece; ^2^ Master Program in “Food, Nutrition and Microbiome”, Laboratory of Hygiene and Environmental Protection, Department of Medicine, Democritus University of Thrace, Alexandroupolis, Greece

**Keywords:** cancer, bladder, urothelial, papillary, management, elderly

## Abstract

This case study presents the diagnostic and therapeutic course of a 72-year-old male patient with a history of high-grade urothelial carcinoma with papillary features. The report outlines the patient’s initial presentation, the intervention strategies employed, including transurethral resection and intravesical Bacillus Calmette-Guérin (BCG) therapy, the subsequent complications and clinical decisions following the intense symptoms post-treatment. The study highlights the challenges in managing bladder cancer in elderly patients, considering the tumor’s characteristics, treatment responses, and the patient’s quality of life.

## 1 Introduction

Globally, bladder cancer ranks as the 10th most diagnosed cancer, with an estimated 573,000 new cases and 213,000 fatalities reported.

The prevalence and death rates from this cancer are significantly higher in males than females, with men experiencing incidence and mortality rates of 9.5 and 3.3 per 100,000, respectively—about four times higher than those in women worldwide.

Among men, bladder cancer is classified as the 6th most frequent cancer and the 9th leading cause of cancer-related death.

The highest incidence rates for bladder cancer are observed in Southern Europe, including Greece with the highest rate among men globally, Western Europe, specifically in Belgium and the Netherlands, and in Northern America. For women, the highest incidence rate globally is recorded in Hungary (1).

The treatment strategies for high-risk urothelial carcinoma of the bladder (UCB), emphasizes a multi-faceted approach like the importance of surgical resection, the role of intravesical therapy (particularly with BCG and chemotherapy) in reducing recurrence and progression, and the potential consideration of radical cystectomy for certain cases (2).

## 2 Case presentation

This case report outlines the diagnostic and therapeutic journey of a 72-year-old male patient diagnosed with high-grade urothelial carcinoma, emphasizing the complexities of managing elderly patients with this condition.

The patient, presenting with hematuria and a history of bladder discomfort, underwent diagnostic evaluations revealing high-grade urothelial carcinoma with papillary features. Histological findings confirmed the diagnosis, indicating a pT1 stage cancer with WHO 2016/2004 grade 2 and 3 malignancy.

## 3 Treatment and outcome

Initial treatment involved transurethral resection of bladder tumors (TURBT), followed by intravesical BCG immunotherapy. He underwent 9 BCG instillations and after completing the 7th instillation, he experienced symptoms like burning and painful urination.

**Figure 1:**
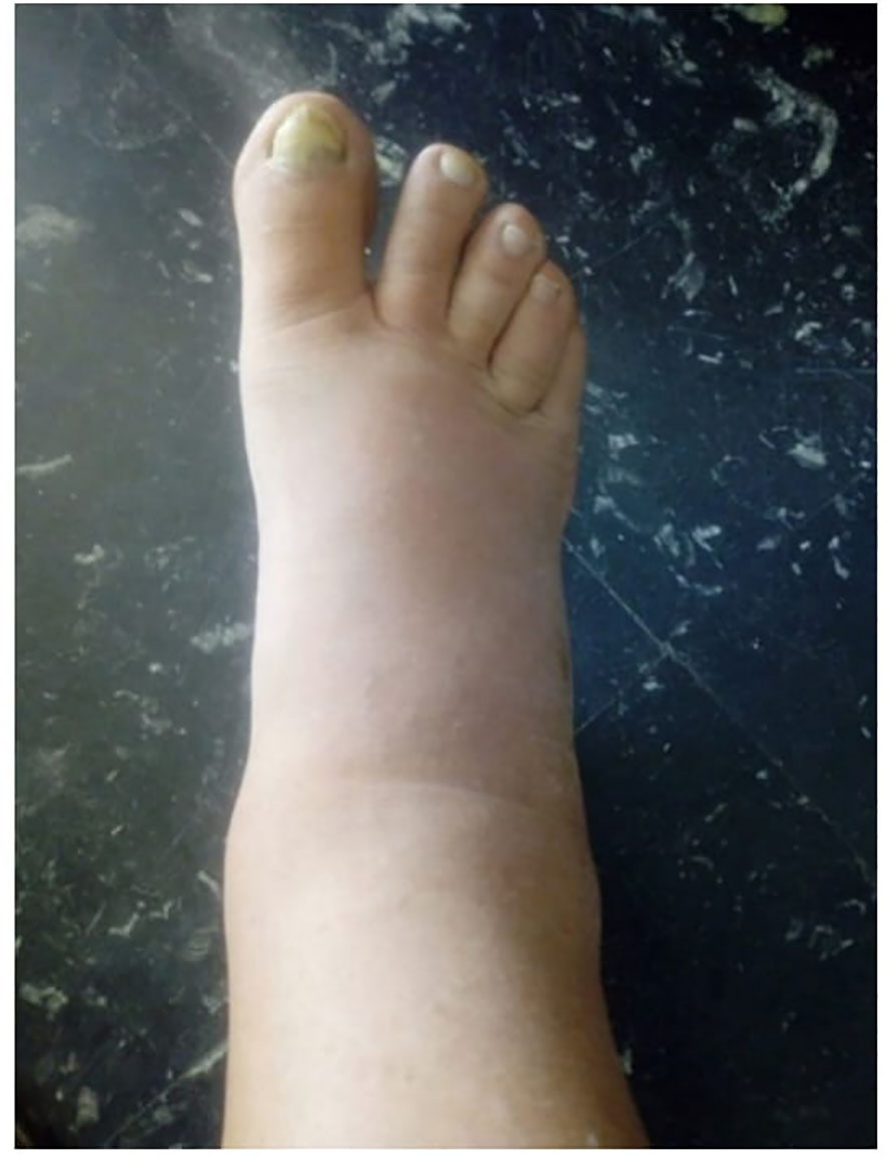
Swelling of patient’s right leg ten days after the 9th instillation.

The patient developed intense symptoms in the lower urinary tract after the 8th BCG instillation, and experienced swelling in the lower extremities ten days after the 9th instillation, which resulted in discontinuation of treatment (see **Figure 1**).

Within six months post-BCG therapy, the patient experienced severe sepsis characterized by a high fever of 39°C, low blood pressure at 68/39 mmHg, confusion, rapid heartbeat, and pronounced hematuria. Elevated white blood cell counts (11900*10^9^/L), PMNs (9740*10^9^/L), Hemoglobin (11 g/dL), MCV (62.4fL), MCH (19.7pg), ESR (64 mm/h), CRP levels (32.171 mg/dL), urea (85mg/dL) and creatinine (3.61 mg/dL) were also noted in laboratory findings. In the microscopic examination of urine, 43-50 pus cells per high-power field, numerous microorganisms, and from aerobic urine culture, 3 types of bacteria were found with the sample being contaminated. Thus, empirical antimicrobial therapy was initiated.

Subsequent treatment with antibiotics (amikacin, ciprofloxacin) alleviated the symptoms and laboratory tests returned to the normal range. Although the acute episode of infection was treated properly, throughout these 6 months, the patient still reported an increased urgency to urinate, approximately every half hour. A following cystoscopy, along with transurethral resection, new biopsy, and electrocoagulation of the visible lesions were conducted. The biopsy result ruled out tuberculous cystitis (Ziehl-Neelsen culture: negative).

An ultrasound encompassing the kidneys, ureters, bladder, and prostate was conducted, revealing a capacity of the bladder at 102.6 cc. The Urologist who was following up the patient, suggested a treatment with solifenacin and mirabegron. The patient received the medication for approximately 3 months which resulted in gradual improvement of symptoms.

Moreover, the patient sought advice by a rheumatologist due to persistent weakness and swelling of the lower limbs. He underwent a thorough investigation, including immunological tests which were all negative, measurement of bone density, MRI of the lumbosacral spine, MRI of the cervical spine and spinal cord, CT of the retroperitoneum, lower abdomen, and pelvic area.

A possible relation with the BCG treatment could not be excluded. With the reason of the arthritis remaining unclear, the patient was prescribed a three-month regimen of prednisolone, dosed at 5 mg per day. After two months, there was a notable improvement of the symptoms.

In 2021, urinary cytology was performed again, with no evidence of malignancy found. In 2022, urine sample from automated urination showed elements consistent with urothelial carcinoma. In 2023, rare, atypical urothelial cells were found (category III according to the Paris system), prompting monitoring and reevaluation. In 2024, the new urine sample was suspicious for high-grade urothelial carcinoma (Category IV according to the Paris system) and nuclear atypia grade II. Histological examination of bladder sections revealed microscopic development of *in situ* carcinoma in one of the samples. Immunohistochemistry showed CK 20+ (intense diffuse positivity), with no evidence of infiltrative malignancy. Urinary cytology material tested positive for malignancy with high-grade morphological characteristics of urothelial carcinoma (TPS V), and cytology material from the right kidney tested positive for malignancy with high-grade morphological characteristics (TBS V), while the left kidney was negative for high-grade urothelial carcinoma. For these reasons, placement of a right ureteral stent-pigtail and BCG instillation were decided upon.

## 4 Discussion

Differentiating the stages of urothelial carcinoma is essential for establishing the correct treatment approach and forecasting the patient’s prognosis. Specifically, the pT1 stage of urothelial carcinoma is characterized by the tumor’s penetration into the lamina propria without extending into the muscularis propria, which is pivotal in guiding therapeutic decisions and assessing the patient’s future health trajectory (3).

Below we can see the staging of primary tumors in bladder cancer (**Table 1**) (4).

**Table 1:** Staging of Primary Tumors (T) in Bladder cancer.

Stage	Description
**TX**	Primary tumor cannot be assessed
**Ta**	Noninvasive papillary carcinoma
**Tis**	Carcinoma *in situ* (CIS)
**T1**	Tumor invades lamina propria
**T2**	Tumor invades muscularis propria
**T2a**	Tumor invades superficial muscularis propria (inner half)
**T2b**	Tumor invades deep muscularis propria (outer half)
**T3**	Tumor invades perivesical tissue/fat
**T3a**	Tumor invades perivesical tissue/fat microscopically
**T3b**	Tumor invades perivesical tissue/fat macroscopically (extravesical mass)
**T4**	Tumor invades prostate, uterus, vagina, pelvic wall, or abdominal wall
**T4a**	Tumor invades adjacent organs (uterus, ovaries, prostate stoma)
**T4b**	Tumor invades pelvic wall and/or abdominal wall

The most frequent initial sign of bladder cancer is hematuria without pain, affecting around 85% of patients. In practice, almost all individuals with bladder tumors detectable through cystoscopy exhibit some level of microhematuria when sufficient urine samples are analyzed (5).

Transurethral resection of the bladder tumor (TURBT) is the primary treatment for Ta and T1 bladder cancers, crucial for diagnosis, staging, and risk assessment. While TURBT alone may suffice for low-grade tumors, high-grade Ta and T1 cancers have significant recurrence rates, necessitating adjuvant intravesical therapies. These therapies, particularly intravesical BCG and chemotherapeutic agents like Mitomycin C, aim to reduce recurrence by targeting residual tumor cells in the bladder, with some strategies including perioperative and maintenance treatments to enhance efficacy and prevent reimplantation of tumor cells (6).

Non-muscle-invasive bladder cancer (NMIBC) represents a varied subgroup of urothelial carcinoma with differing risks of recurrence and progression to muscle-invasive stages. Guidelines from the American Urological Association (AUA) and the European Association of Urology (EAU), as well as predictive tools like nomograms based on clinical data, are used for risk assessment to guide treatment, though they may not be precise for every case. Post-surgical (after TURBT) adjuvant treatments, including intravesical therapy, are crucial for managing the disease while considering side effects. Chemotherapy and BCG intravesical treatments are widely researched and result in favorable outcomes for many, but still, a significant number of patients experience recurrence within two years, and some progress to muscle-invasive cancer. This underscores the growing need for new therapies that lessen the treatment burden. The current environment of NMIBC and the reasoning for new chemoablative treatments before surgery are examined in this context.

**Figure 2:**
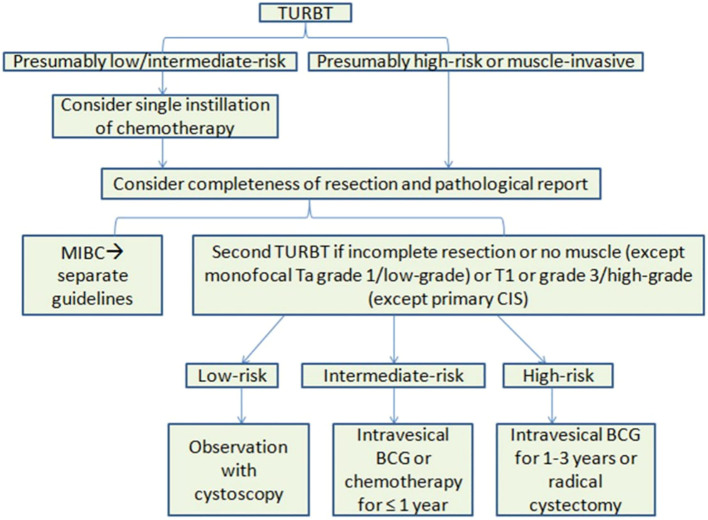
Overview of EAU treatment guidelines for NMIBC. BCG, Bacillus Calmette-Guérin; CIS, carcinoma in situ; EAU, European Association of Urology, MIBC, muscle-invasive bladder cancer; NMIBC, non-muscle-invasive bladder cancer; TURBT, transurethral resection of bladder tumor.

The standard of care for initial treatment of bladder lesions includes TURBT followed by intravesical chemotherapy or BCG, based on the patient’s risk. For low to intermediate risk patients, a single chemotherapy instillation post-TURBT is usual. After confirming risk levels, monitoring or intravesical chemotherapy is advised for low-risk, while BCG is generally for high-risk in the initial setting, with intermediate-risk patients also considered. Recent BCG shortages have led to recommendations to reserve BCG for high-risk patients, with intravesical chemotherapy as a first-line alternative for intermediate-risk patients. Induction BCG involves weekly instillations for six weeks, with some intermediate/high-risk patients also receiving extended maintenance therapy, though there’s no agreement on its length. In **Figure 2**, it is presented the treatment guidelines for NMIBC (7).

BCG (Bacillus Calmette-Guérin) is a key treatment for intermediate and high-risk non-muscle invasive bladder cancer (NMIBC), effectively reducing recurrence and progression rates. While its exact mechanism remains under study, BCG appears to trigger both innate and adaptive immune responses, leading to tumor destruction. The treatment involves an initial induction phase and, often, a maintenance phase to enhance efficacy. Despite its success, BCG treatment can have side effects, though serious complications are rare. Research continues to refine understanding and application of BCG in bladder cancer treatment (8).

The best approach for treating patients with high-grade non-muscle-invasive bladder cancer (NMIBC) that persists or recurs after two rounds of BCG therapy (such as two 6-week induction courses or one 6-week induction course plus a 3-week maintenance course) and who cannot or choose not to have a cystectomy is still unclear. Ongoing research into new treatments for these patients is crucial. Healthcare providers are encouraged to look for clinical studies and consider enrolling eligible patients in these trials (4).

These systemic complications are relatively rare and result from BCG dissemination into the bloodstream, affecting various organ systems such as musculoskeletal, vascular, pulmonary, hepatic, lymphatic, sepsis and other complications. These complications are rare but can be serious, requiring immediate diagnosis and treatment (9).

Recently, pembrolizumab was FDA-approved for intravenous use, based on preliminary data from the Keynote-057 study, a Phase II multicenter study with a single-arm design (10).

Pembrolizumab, a programmed cell death protein (PD-1) inhibitor, has shown promise in treating bladder cancer, particularly in cases with high programmed death-ligand 1 (PD-L1) expression and relapsed tumors, with the Keynote-057 study revealing a 17% rate of lasting complete response after 12 months (11).

This approval of pembrolizumab, based on findings by Balar et al., endorses it as an effective non-surgical treatment choice for patients with BCG-unresponsive carcinoma *in situ* of the bladder who cannot undergo or choose not to have radical cystectomy. It addresses a significant gap in available treatments and sets a precedent for future research aimed at achieving improved and longer-lasting outcomes for patients with BCG-unresponsive carcinoma *in situ*, whether with or without papillary tumors (12).

Many other treatments are currently under investigation for patients with BCG-unresponsive NMIBC. Findings from a phase 2/3 multicenter trial showed that nogapendekin alfa inbakicept (NAI, also known as N-803), an IL-15 superagonist, exhibits a significant synergistic effect when administered alongside BCG for this condition. This therapy was granted FDA approval in April 2024 (13).

The measurement of quality of life indicates that the intravesical combination of nogapendekin alfa inbakicept (NAI) and BCG is well-tolerated by patients with high-grade NMIBC that is unresponsive to BCG, particularly those with carcinoma *in situ* (CIS). These findings further support the positive benefit-risk profile of this innovative combination immunotherapy for a difficult-to-treat condition (14).

This case emphasizes the need for individualized treatment plans in elderly patients, considering the balance between therapeutic efficacy and quality of life. It underscores the importance of meticulous follow-up to manage and mitigate treatment-induced complications.

The patient’s treatment for high-grade urothelial carcinoma included transurethral resection and BCG immunotherapy. Despite these interventions, the patient experienced significant complications, including severe lower urinary tract symptoms (LUTS) that necessitated the discontinuation of BCG therapy, and a septic episode characterized by hypotension, fever, and elevated white blood cells, which responded to antibiotics. These complications highlight the challenges in managing such cases, especially in elderly patients, balancing the aggressiveness of treatment against potential side effects and the patient’s overall quality of life. The decision-making process was guided by the need to manage the cancer effectively while minimizing harm, underscoring the importance of personalized care strategies in this demographic.

The detailed laboratory findings and continuous monitoring of the patient’s condition are consistent with the predictive approaches and emphasis on CRP levels highlighted in other studies (15, 16). High preoperative CRP levels and low hemoglobin levels are independent prognostic factors linked to poor outcomes in patients undergoing radical cystectomy for urothelial carcinoma of the bladder. Additionally, the TNR-C score, which consists of CRP levels, pathological T score, lymph node density, resection margins, age, and tumor size, was validated in a large cohort of patients. The use of these routine biomarkers is proposed for individual risk stratification and optimization of therapeutic strategies in these patients (17).

Based on the details provided in this case study, the elderly patient belongs to a high-risk category for urothelial carcinoma of the bladder, necessitating a personalized and cautious approach to treatment. Although the patient did not undergo cystectomy, his high preoperative CRP levels (32.171 mg/dL) and low hemoglobin levels (11 g/dL) are indicators of a poor prognosis, aligning with the findings of this study. Given the patient’s high CRP levels, he would likely score high on the TNR-C scale, indicating a higher risk and the need for tailored treatment strategies to manage his condition effectively. Overall, the elderly patient’s case fits well within the frameworks of these studies, highlighting the importance of using prognostic biomarkers and routine laboratory parameters to optimize outcomes and manage risks effectively.

## 5 Follow-up and long-term implications NMIBC

There isn’t a universally agreed-upon protocol for post-treatment surveillance due to the reliance on data from retrospective studies. Thus, the scheduling and duration of follow-up procedures like cystoscopy and additional imaging are tailored to match each patient’s specific risk level for recurrence and progression. For those newly diagnosed with Ta-T1 tumors and/or CIS, it’s advised to perform the initial cystoscopy at three-month intervals. It’s then recommended to continue regular cystoscopy and cytology checks every three to six months for the first two years, and every six to twelve months afterwards. For those with high-risk tumors, routine imaging of the upper urinary tract, such as CT urography, is suggested (18).

## 6 Conclusion

Treating high-grade urothelial carcinoma in older patients necessitates a tailored strategy that balances the advantages and potential risks of available treatments. More studies are needed to refine the approaches for managing this patient group. The goal of this analysis is to enrich the current knowledge base by underlining the unique obstacles and factors in managing high-grade urothelial carcinoma in older adults, emphasizing the need for individualized treatment plans.

## Data availability statement

The original contributions presented in the study are included in the article/supplementary material. Further inquiries can be directed to the corresponding author.

## Ethics statement

Written informed consent was obtained from the individual(s) for the publication of any potentially identifiable images or data included in this article.

## Author contributions

CN: Conceptualization, Data curation, Formal analysis, Writing – original draft. DG: Data curation, Formal analysis, Investigation, Writing – original draft. CA: Investigation, Methodology, Project administration, Writing – review & editing. ES: Investigation, Supervision, Validation, Writing – review & editing.
